# Causal Effect of Chondroitin, Glucosamine, Vitamin, and Mineral Intake on Kidney Function: A Mendelian Randomization Study

**DOI:** 10.3390/nu15153318

**Published:** 2023-07-26

**Authors:** Jeong-Min Cho, Jung-Hun Koh, Seong-Geun Kim, Soojin Lee, Yaerim Kim, Semin Cho, Kwangsoo Kim, Yong-Chul Kim, Seung-Seok Han, Hajeong Lee, Jung-Pyo Lee, Kwon-Wook Joo, Chun-Soo Lim, Yon-Su Kim, Dong-Ki Kim, Sehoon Park

**Affiliations:** 1 Department of Internal Medicine, Seoul National University Hospital, Seoul 03080, Republic of Korea; als6494@naver.com (J.-M.C.);; 2 Department of Internal Medicine, Inje University Sanggye Paik Hospital, Seoul 01757, Republic of Korea; 3 Department of Internal Medicine, Uijeongbu Eulji University Medical Center, Uijeongbu 11759, Republic of Korea; 4 Department of Internal Medicine, Seoul National University College of Medicine, Seoul 07061, Republic of Korea; 5 Department of Internal Medicine, Keimyung University School of Medicine, Daegu 42601, Republic of Korea; 6 Department of Internal Medicine, Chung-Ang University Gwangmyeong Hospital, Gwangmyeong 14353, Republic of Korea; 7 Transdisciplinary Department of Medicine & Advanced Technology, Seoul National University Hospital, Seoul 03080, Republic of Korea; 8 Kidney Research Institute, Seoul National University, Seoul 03080, Republic of Korea; 9 Department of Internal Medicine, Seoul National University Boramae Medical Center, Seoul 07061, Republic of Korea; 10 Department of Biomedical Sciences, Seoul National University College of Medicine, Seoul 03080, Republic of Korea

**Keywords:** chondroitin, glucosamine, vitamins, minerals, glomerular filtration rate

## Abstract

The causal effects of chondroitin, glucosamine, and vitamin/mineral supplement intake on kidney function remain unknown, despite being commonly used. We conducted a two-sample summary-level Mendelian randomization (MR) analysis to test for causal associations between regular dietary supplement intake and kidney function. Genetic instruments for chondroitin, glucosamine, and vitamin/mineral supplement intake were obtained from a genome-wide association study of European ancestry. Summary statistics for the log-transformed estimated glomerular filtration rate (log-eGFR) were provided by the CKDGen consortium. The multiplicative random-effects inverse-variance weighted method showed that genetically predicted chondroitin and glucosamine intake was causally associated with a lower eGFR (chondroitin, eGFR change beta = −0.113%, standard error (SE) = 0.03%, *p*-value = 2 × 10^−4^; glucosamine, eGFR change beta = −0.240%, SE = 0.035%, *p*-value = 6 × 10^−12^). However, a genetically predicted vitamin/mineral supplement intake was associated with a higher eGFR (eGFR change beta = 1.426%, SE = 0.136%, *p*-value = 1 × 10^−25^). Validation analyses and pleiotropy-robust MR results for chondroitin and vitamin/mineral supplement intake supported the main results. Our MR study suggests a potential causal effect of chondroitin and glucosamine intake on kidney function. Therefore, clinicians should carefully monitor their long-term effects.

## 1. Introduction

A kidney is an organ that metabolizes various drugs through enzyme systems and excretes the metabolites from the body via glomerular filtration and tubular secretion [1]. The formation of potential nephrotoxic metabolites due to drug ingestion and their concentration in the kidney medulla and interstitium may lead to drug-induced nephrotoxicity [2]. As the general population is largely exposed to various over-the-counter drugs [3,4], it is important to identify the effects of these drugs on kidney function.

Chondroitin and glucosamine are major structural components of cartilage used as symptomatic slow-acting drugs for both osteoarthritis (OA) and joint discomfort [5]. These glycosaminoglycan precursors have been widely used as over-the-counter drugs or dietary supplements because of their tolerability and safety [6,7,8]. Chondroitin and glucosamine are associated with a reduction in joint pain, improvement of joint function, and reduction in joint space narrowing in patients with OA [6,7,9,10]. Despite a protective effect against various pathological conditions, including inflammation [11,12], cardiovascular disease [13], and diabetes [14], there have been some reports of non-specific kidney injury and toxicity associated with these chondroprotective drugs [15,16,17]. Nevertheless, the causal association between chondroitin or glucosamine intake and kidney function has not been established yet.

Mendelian randomization (MR) is a statistical method to evaluate the causal association between exposure and outcome traits using genetic instruments that are closely linked to exposure [18]. As MR analysis uses a genetic instrument fixed at conception, the causal association is less susceptible to confounding and reverse causality. Thus, MR has been widely used to demonstrate causal linkages between complex phenotypes, including kidney function traits, in several epidemiological studies recently [19,20,21].

In the present study, we performed an MR analysis to identify the causal effects of various dietary supplements, including chondroitin, glucosamine, and vitamins, on kidney function. We hypothesized that chondroitin and glucosamine intake may be associated with decreased kidney function.

## 2. Materials and Methods

### 2.1. Ethical Considerations

This study was approved by the Institutional Review Board (IRB) of the Seoul National University Hospital (IRB number: E-2306-031-1437). The summary statistics of the kidney function traits are from the public domain (http://ckdgen.imbi.uni-freiburg.de/, accessed on 8 December 2022) of the CKDGen consortium. The requirement for informed consent was waived because this study analyzed anonymous public data and summary statistics.

### 2.2. Study Setting

**Figure 1:**
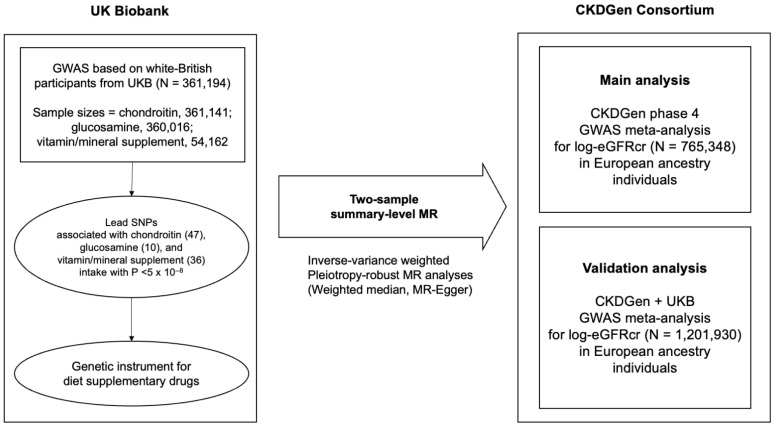
Study flow diagram. The genetic instruments for genetically proxied chondroitin, glucosamine, and vitamin/mineral intake were obtained from GWAS based on white British participants from UKB (*n* = 361,194). The numbers of lead SNPs for chondroitin, glucosamine, and vitamin/mineral supplement intake are described in brackets. Two-sample summary-level MR was conducted on three datasets for kidney function traits, including main analysis implemented on CKDGen phase 4 GWAS meta-analysis for log-eGFRcr (*n* = 765,348). CKDGen is a genetic database for kidney function traits which is publicly available. GWAS = genome-wide association study; UKB = UK Biobank, SNP = single nucleotide polymorphism; MR = Mendelian randomization; eGFR = estimated glomerular filtration rate; log-eGFRcr = creatinine-based log-transformed eGFR.

This was a two-sample summary-level MR study that analyzed the causal effects of dietary supplement intake on kidney function (Figure 1). Genetic instruments for each dietary supplement, including chondroitin, glucosamine, and vitamin supplements, were obtained from a genome-wide association study (GWAS) using UK Biobank (UKB) data, which is a large prospective cohort of over 500,000 enrolled participants. Summary statistics for kidney function were provided by the CKDGen Consortium, which is currently the largest GWAS meta-analysis database of kidney function traits. Both exposure and outcome summary-level data were obtained from high-powered European population-based databases, as two-sample MR analysis requires samples from the same ethnicity to avoid the different SNP-exposure or SNP-outcome association due to different patterns of linkage disequilibrium [18].

### 2.3. Data Sources for Chondroitin, Glucosamine, and Vitamin Supplement Intake

Three sets of genetic instruments were introduced for the intake of chondroitin, glucosamine, and vitamin and/or mineral supplements (e.g., vitamin C, multivitamin, fish oil, calcium supplement), respectively, from the previous GWAS (Supplemental Table S1) [22]. The GWAS result was released by the Neale Lab (http://www.nealelab.is/uk-biobank, accessed on 1 August 2018) based on 361,194 white British participants from the UKB and was used to establish genetic instruments that were used in the previous MR analysis [22]. The dietary supplement intake data were collected using detailed electronic questionnaires by UKB. Participants were presented with a list of supplements, including chondroitin, glucosamine, and vitamin/mineral supplements, and asked the question, “Do you usually take any of the following?”. A binary classification for regular supplement intake was established based on the questionnaire data (1 = yes; 0 = no). Each phenotype had a sample size of 361,141 participants taking chondroitin, 360,016 participants taking glucosamine, and 54,162 participants taking vitamin and/or mineral supplements. The selected SNPs in each GWAS reached the genome-wide significance threshold of *p* < 5 × 10^−8^ (5 × 10^−6^ for vitamin supplements) and a window of 10,000 kb (r^2^ < 0.001) to confirm their independence. The instrumental strengths were assessed with F-statistics demonstrated of ≥10 [22], indicating a relatively low risk of weak instrumental bias in MR analysis. To identify potential pleiotropic confounders with an association (*p* < 5 × 10^−8^), we screened each instrument SNP in PhenoScanner (http://www.phenoscanner.medschl.cam.ac.uk/, accessed on 6 June 2023) [23]. The average weekly beer plus cider intake, mineral and other dietary supplements (fish oil), and educational status were potential confounders (Supplemental Table S2).

### 2.4. Data Sources for Kidney Function Traits

Summary statistics for kidney function traits were obtained from the CKDGen, currently the largest GWAS meta-analysis database for various kidney function traits of European ancestry [24]. The main MR analysis for kidney function was conducted using the CKDGen meta-analysis for log-transformed creatinine-based estimated glomerular filtration rate (log-eGFRcr) from 2019 (*n* = 765,348) [24]. In the clinical field, estimated glomerular filtration rate (eGFR) is used as a standard parameter for evaluating kidney function as recommended by Kidney Disease: Improving Global Outcome (KDIGO), a global organization for establishing clinical practice guidelines for kidney disease [25]. As data sources for diet supplementary drugs were provided by UKB, we used a CKDGen-based dataset for the main analysis to avoid the potential weak instrumental bias raised by the participants’ overlap [26]. For validation, CKDGen and UKB meta-analyses for log-eGFRcr (*n* = 1,201,930) were used because they were the largest meta-analyses of eGFR despite the possibility of partial overlap of the population among exposure and outcome datasets [27].

### 2.5. MR Assumptions

A valid causal inference may be established through the assessment of three key MR assumptions regarding instrumental variables [18]. First, the relevance assumption indicates that the instrumental variable is strongly associated with exposure. This was established by using genetic instruments from the previous GWAS that met genome-wide significance at a threshold of *p* < 5 × 10^−8^ (except for vitamin supplements intake for *p* < 5 × 10^−6^) and had F-statistics ≥ 10. Second, the independence assumption is that the instrumental variable is not associated with any confounder that affects the outcome. Third, the exclusion restriction assumption is that the IV is associated with the outcome only through exposure. For the second and third assumptions, we performed pleiotropy-robust MR analyses to support the robustness of the inverse-variance weighted (IVW) estimates, even when some of the genetic variants were invalid [28,29]. For example, the weighted median method can provide valid estimates when half of the weight contributed by genetic variants has pleiotropic effects. MR-Egger provides valid estimates, even when all genetic variants have a pleiotropic effect. In addition, we ascertained that none of the instrumental variables violated the direction of causal effects toward exposure, as they did not exhibit a direct effect (*p* < 5 × 10^−8^) on the outcome.

### 2.6. Two-Sample Summary-Level MR Analysis

The multiplicative random-effects IVW method was performed as the main MR analysis, as recommended in the guidelines for MR analysis [18]. The multiplicative random-effects IVW provides valid causal estimates under the assumption of balanced pleiotropy. Pleiotropy-robust MR methods, including MR-Egger [28] and weighted median [29], have been implemented to address valid causal estimates, even under conditions of unbalanced pleiotropy. Single-SNP and leave-one-out analyses were performed to identify the presence of a disproportionate effect of one SNP. Cochran’s Q test was used to identify heterogeneity. In addition, as there were some potential confounders found by PhenoScanner, we performed a sensitivity analysis by consecutively excluding groups of SNPs that were involved in the same phenotype to establish the robustness of our findings regardless of the effect of the potential confounders.

The summary-level MR analysis was performed by the “TwoSampleMR” package (Version 0.4.26). Regarding the issue of multiple comparisons, as the finding that was consistent throughout the study outcome and sensitivity analysis was reported to be significant, we used the conventional two-sided *p* < 0.05 as the threshold level of significance.

## 3. Results

### 3.1. Characteristics of the Data Sources

Summary statistics of the exposure and outcome data were obtained from the UKB and CKDGen. UKB is a population-based prospective cohort study that included over 500,000 Europeans aged between 40 and 69 years between 2009 and 2010 [30]. The average age was 56.5 ± 8.1, and 45.6% were male. The prevalence of CKD was 7.4%, and the mean serum creatinine was 0.82 ± 0.21 mg/dL. The median age of CKDGen GWAS meta-analysis participants was 54 years, and 50% were female. The median of the mean eGFR was 89 (interquartile range, 81–94) mL/min/1.73 m^2^ [24].

### 3.2. MR Analysis of Dietary Supplement Intake on Kidney Function

**Figure 2:**
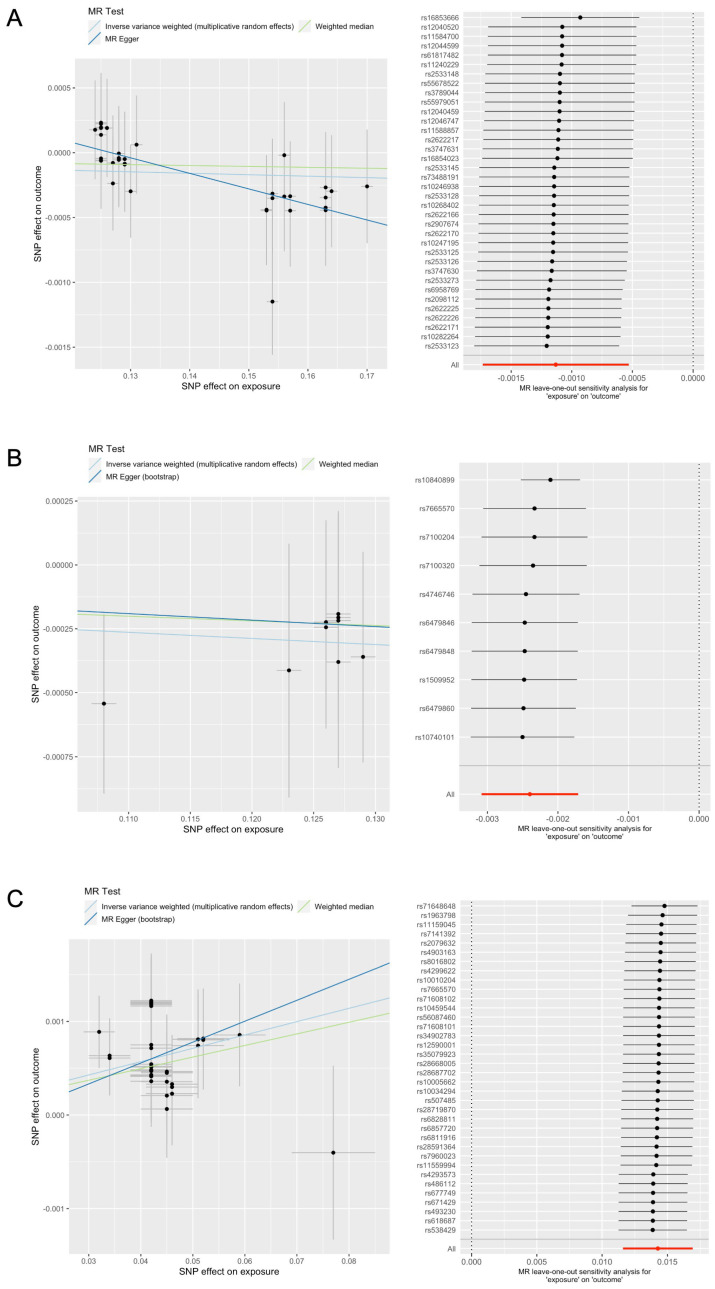
Mendelian randomization plots for causal association between (**A**) chondroitin, (**B**) glucosamine, and (**C**) vitamin/mineral supplement intake and kidney function. For each exposure, left and right plots show scatter and leave-one-out plots, respectively. Scatter plot of the effect sizes of the association between SNP-genetically predicted intake of each dietary supplement (x-axis) against the SNP-log-eGFRcr (y-axis) with standard error bars. The slopes of the lines correspond to the causal estimates per method. Each black point of the leave-one-out analysis represents the MR-IVW method applied to estimate the causal effect of genetically predicted intake of each dietary supplement on kidney function, excluding that certain variant from the analysis. The red point indicates the IVW estimate using all SNPs. There were no instances where the exclusion of a single SNP led to dramatic changes in the overall result.

In the summary-level MR analysis, the IVW results showed a significant association between genetically predicted regular chondroitin, vitamin/mineral supplements, and glucosamine intake and kidney function (Table 1, Figure 2). In the main analysis, genetically predicted chondroitin and glucosamine intake were significantly associated with lower eGFR. In contrast, genetically predicted vitamin/mineral supplement intake was significantly associated with a higher eGFR. The validation analyses of chondroitin and vitamin/mineral supplement intake showed results concordant with those of the main analyses. In other words, regular chondroitin intake was associated with a lower eGFR, whereas regular vitamin/mineral intake and kidney function were associated with a higher eGFR in different outcome datasets. Regarding glucosamine intake, however, the results of the main analyses were not replicated when analyzed using the log-eGFRcr meta-analysis summary statistics provided by the CKDGen and UKB.

The pleiotropy-robust MR analyses (Supplemental Table S3), using genetically predicted chondroitin and vitamin/mineral supplement intake as exposures, supported the main findings, whereas the results from glucosamine intake did not align with the main MR results. The leave-one-out analysis demonstrated no notable outlier effects in the MR analyses conducted with the three exposures.

### 3.3. Sensitivity Analysis for Chondroitin Intake

Regarding the MR results for chondroitin intake and kidney function, an MR-Egger intercept *p* < 0.05 was observed, indicating potential pleiotropy. Therefore, we conducted additional sensitivity analyses using PhenoScanner to identify potential confounders (Supplemental Table S4). First, we examined the association between genetically predicted chondroitin intake and kidney function after excluding the SNPs associated with the phenotype “beer plus cider intake” and identified that the significant inverse association between chondroitin intake and kidney function was maintained. Furthermore, we assessed the causal relationship between chondroitin intake and kidney function after excluding the SNPs associated with “high educational status” and found that the results were similar to the main finding. Specifically, although the MR estimates from sensitivity analysis using the main dataset showed no significant association between chondroitin intake and eGFR, the results from the validation dataset showed a significant association between genetically predicted chondroitin intake and lower eGFR.

## 4. Discussion

In this MR study, we identified causal linkages between the intake of over-the-counter drugs, including chondroitin, glucosamine, and vitamin/mineral supplements, and kidney function. Chondroitin was significantly associated with a lower eGFR, whereas vitamin/mineral supplement intake was associated with a higher eGFR. Although glucosamine intake was causally associated with a lower eGFR in the main analysis, the results were not replicated in the validation datasets. Our study suggests that chondroitin intake may decrease kidney function, whereas vitamin and mineral supplement intake may preserve kidney function.

Chondroitin and glucosamine sulfate are widely used over-the-counter supplementary drugs for relieving joint pain and delaying the progression of OA because of their safety profile and tolerability [6,7,8,31,32]. These chondroprotective drugs are commonly used as dietary supplements, even among patients who are at high risk of kidney function decline, including kidney donors [33]. Chondroitin and glucosamine are absorbed through the gastrointestinal tract, metabolized mostly in the liver, and eliminated via the kidneys. When intravenously injected, more than 30% of the glucosamine sulfate is excreted in urine [34]. However, there is only limited data regarding the metabolism, excretion, and toxicity of chondroitin and glucosamine. Furthermore, studies examining the potential effects of these drugs on the kidneys are lacking.

Our study has several strengths. First, we conducted an MR analysis that provided causal estimates between supplementary drug intake and kidney function. These results are less prone to confounding and are unaffected by reverse causation, both of which are limitations of observational studies. Second, this study provided valuable data on the adverse causal effects of regular chondroitin intake on kidney function. Our findings have clinical implications and suggest that patients taking chondroprotective drugs should be monitored for changes in kidney function. Additionally, patients who are at high risk of kidney function impairment should be better informed of potential kidney function impairment before the ingestion of such drugs. As the majority of patients with bone or joint disease who are likely to be ingesting chondroprotective drugs share similar clinicodemographic characteristics with those at higher risk of kidney injury, additional attention to this population is needed [35].

In the present study, we identified the causal effect of chondroitin intake on reduced kidney function using MR analysis. Several randomized placebo-controlled trials for chondroitin and glucosamine did not exhibit adverse effects related to kidney function [8,10,32,36]. However, MR analysis tests the lifetime effect of a genetically predicted modifiable exposure on an outcome; thus, our study suggests that long-term kidney effects from such substance use may be clinically significant. In this regard, our findings suggest that clinicians should carefully monitor long-term chondroprotective agent use, particularly in patients at high risk of kidney function impairment. Our results are supported by previous reports that raised concerns regarding the nephrotoxic effects of chondroprotective drugs and their impact on kidney function [15,16,17]. A few patients who regularly ingested glucosamine exhibited a decline in kidney function without any other precipitating factors, and acute or chronic tubulointerstitial nephritis was confirmed in kidney biopsies. Following the discontinuation of glucosamine, the eGFR of patients with glucosamine-related tubulointerstitial nephritis showed some improvement; however, their kidney function did not fully recover to baseline, and they remained in chronic kidney disease status. In an experimental study, glucosamine-related nephrotoxicity has been suggested to be associated with apoptosis in kidney tubular and mesangial cells with overexpression of transforming growth factor-β and connective tissue growth factor [17]. As these factors are responsible for mesangial and tubulointerstitial fibrosis, it is possible that chondroprotective drug-related kidney injuries may involve an irreversible component.

The anti-inflammatory and antioxidative properties of various vitamins and minerals that prevent kidney injury have been proposed in previous experimental studies [37,38,39,40,41,42]. Our findings are in line with those of previous studies, suggesting a potential kidney-protective effect of ingesting regular vitamin/mineral supplements in the general population. Nevertheless, further interventional studies are needed to identify the effect and an adequate dose for preserving kidney function, as the current MR analysis relies on self-reporting of vitamin/mineral supplement intake with varying product content and quality.

The current study had some limitations. First, the regular use of dietary supplements was ascertained by self-reporting at baseline. In addition, the details of drug intake, including dosage, components, and duration, were not provided, thus weakening the study findings. Second, there might be a possibility of weak instrumental bias in validation analyses, as some of the exposure and outcome samples obtained from the UKB may overlap [26]. The discrepancies in MR results for glucosamine intake in the main and replication analyses may be explained by this; thus, the possibility of a false negative may not be excluded. Third, because genetic instruments reflect lifetime exposure, transient changes in a higher degree of supplementary drug intake may have a greater impact on kidney function. Fourth, for UKB and CKDGen, the study population was mainly white Europeans; therefore, generalizability was not identified for other ethnicities, and validation analyses should be performed to ascertain the causal effects between diet supplementary drug intake and kidney function in other ethnicities.

## 5. Conclusions

This MR study suggests that regular chondroitin intake reduces eGFR. Clinicians should understand the potential adverse effects of chondroprotective drugs on kidney function and consider regular monitoring of laboratory parameters. Further studies are warranted to identify the underlying mechanisms of chondroitin and glucosamine intake and impairment of kidney function.

## Figures and Tables

**Table 1:** Summary-level MR analysis of dietary supplement intake and kidney function.

Exposure	Analysis	^a^ Outcome	eGFR Change Beta (%)	Standard Error (%)	*p*-Value
Chondroitin	Main	Creatinine-based log-eGFR (CKDGen)	−0.113	0.030	2 × 10^−4^
	Validation	Creatinine-based log-eGFR (CKDGen + UKB)	−0.283	0.016	4 × 10^−31^
Glucosamine	Main	Creatinine-based log-eGFR (CKDGen)	−0.240	0.035	6 × 10^−12^
	Validation	Creatinine-based log-eGFR (CKDGen + UKB)	0.424	0.091	3 × 10^−6^
Vitamin/ mineral supplement intake	Main	Creatinine-based log-eGFR (CKDGen)	1.426	0.136	1 × 10^−25^
	Validation	Creatinine-based log-eGFR (CKDGen + UKB)	1.259	0.141	4 × 10^−19^

MR = Mendelian randomization; eGFR = estimated glomerular filtration rate. All MR results are calculated by multiplicative random-effects inverse-variance weighted method. ^a^ For main and validation datasets, meta-analysis of creatinine-based log-eGFR from CKDGen and meta-analysis of creatinine-based log-eGFR from the CKDGen and UKB were used as outcome summary statistics, respectively [24,27]. The MR estimates were converted into the degree of change (percentage [standard error]) of log-transformed eGFR to facilitate interpretation.

## Data Availability

All data used in this work are presented in the Supplemental Materials that accompany the manuscript and are available in the original publications. The data used in this study are publicly available on the consortium website of The CKDGen (URL: https://ckdgen.imbi.uni-freiburg.de/, accessed on 8 December 2022).

## Supplementary Materials

The following supporting information can be downloaded at: https://www.mdpi.com/article/10.3390/nu15153318/s1, Table S1: Genetic instruments of chondroitin, glucosamine, and vitamin and/or mineral supplement intake used for the Mendelian randomization analysis; Table S2: Potential confounders of SNPs for chondroitin intake under the condition of *p* < 5 × 10^−8^ in the PhenoScanner; Table S3: Pleiotropy-robust MR results for dietary supplement intake and kidney function; Table S4: Sensitivity analyses of chondroitin intake and kidney function after exclusion of SNPs associated with potential confounders identified in PhenoScanner.
