# Supplementary material for: Causal Effect of Chondroitin, Glucosamine, Vitamin, and Mineral Intake on Kidney Function: A Mendelian Randomization Study
Source: Nutrients. 2023 Jul 26;15(15):3318. doi: 10.3390/nu15153318 (PMC10421197; doi:10.3390/nu15153318)
Supplement: Supplementary file 1 [file nutrients-15-03318-s001.zip › nutrients-2497463-supplementary.pdf]

Supplemental Materials Table of Contents

Supplemental Table S1. Genetic instruments of chondroitin, glucosamine, and vitamin and/or mineral supplement intake used for the Mendelian randomization analysis

Supplemental Table S2. Potential confounders of SNPs for chondroitin intake under the condition of $P < 5 \times 10^{-8}$ in the PhenoScanner

Supplemental Table S3. Pleiotropy-robust MR results for dietary supplement intake and kidney function

Supplemental Table S4. Sensitivity analyses of chondroitin intake and kidney function after exclusion of SNPs associated with potential confounders identified in PhenoScanner

Supplemental References

Supplemental Table S1. Genetic instruments of chondroitin, glucosamine, and vitamin/mineral supplement intake used for the Mendelian randomization analyses

SNP	CHR	POS	Effect allele	Other allele	Beta	SE	P-value
**Chondroitin**							
rs10238899	7	153492868	C	T	-0.128	0.001	2.81E-08
rs10246938	7	153466423	T	A	-0.129	0.001	1.31E-08
rs10247195	7	153466623	G	A	-0.129	0.001	1.34E-08
rs10263816	7	153470742	G	A	-0.129	0.001	1.28E-08
rs10268402	7	153485627	C	T	-0.125	0.001	3.73E-08
rs10282264	7	153492809	T	C	-0.125	0.001	3.97E-08
rs11240229	1	204576522	T	C	0.163	0.001	5.55E-09
rs11584700	1	204576983	G	A	0.163	0.001	5.23E-09
rs11588857	1	204587047	A	G	0.164	0.001	4.16E-09
rs12040459	1	204564558	A	G	0.157	0.001	2.71E-08
rs12040520	1	204564762	A	G	0.153	0.001	4.31E-08
rs12044599	1	204564714	G	A	0.153	0.001	4.18E-08
rs12046747	1	204593696	A	G	0.154	0.001	2.02E-08
rs16853666	1	204403856	C	T	0.154	0.001	3.30E-08
rs16854023	1	204551830	C	T	0.170	0.001	5.47E-09
rs2098112	7	153487944	A	G	-0.124	0.001	4.19E-08
rs2159462	7	153478989	A	G	-0.127	0.001	2.49E-08
rs2533123	7	153483021	G	A	-0.125	0.001	3.61E-08
rs2533125	7	153473188	G	A	-0.128	0.001	1.81E-08
rs2533126	7	153473086	A	G	-0.128	0.001	1.81E-08
rs2533128	7	153471543	G	A	-0.127	0.001	2.07E-08
rs2533133	7	153466068	A	G	-0.129	0.001	1.47E-08
rs2533137	7	153465680	C	G	-0.128	0.001	1.74E-08
rs2533145	7	153463438	G	T	-0.129	0.001	1.29E-08
rs2533148	7	153462806	C	T	-0.130	0.001	1.11E-08
rs2533196	7	153489530	A	G	-0.128	0.001	2.85E-08
rs2533273	7	153485282	A	C	-0.131	0.001	9.57E-09
rs2538462	7	153497853	C	T	-0.137	0.001	3.73E-08
rs2622103	7	153496171	A	G	-0.138	0.001	1.28E-08
rs2622166	7	153486905	A	G	-0.125	0.001	4.10E-08
rs2622170	7	153486033	A	T	-0.125	0.001	3.83E-08
rs2622171	7	153485893	G	A	-0.126	0.001	3.20E-08
rs2622185	7	153472469	G	A	-0.129	0.001	1.33E-08
rs2622217	7	153464429	C	G	-0.127	0.001	2.05E-08
rs2622225	7	153492123	G	A	-0.125	0.001	4.02E-08
rs2622226	7	153491516	G	A	-0.125	0.001	3.55E-08
rs2907674	7	153471050	G	A	-0.128	0.001	1.71E-08
rs3747630	1	204588140	A	G	0.156	0.001	4.53E-09
rs3747631	1	204587569	C	G	0.163	0.001	5.01E-09
rs3789044	1	204589101	A	G	0.154	0.001	2.09E-08
rs55678522	1	204573481	A	G	0.163	0.001	5.48E-09
rs55979051	1	204567041	G	A	0.156	0.001	3.33E-08
rs59883612	7	153484125	C	T	-0.127	0.001	3.99E-08
rs60009449	7	153489996	C	T	-0.129	0.001	3.32E-08
rs61817482	1	204570347	A	G	0.157	0.001	2.63E-08

rs6958769	7	153487814	T	C	-0.125	0.001	4.14E-08
rs73488191	7	153465730	G	A	-0.129	0.001	1.43E-08
**Glucosamine**							
rs10740101	10	64746142	G	A	0.127	0.001	4.42E-08
rs10840899	12	17996887	A	G	-0.108	0.001	4.75E-08
rs1509952	10	64739668	T	C	0.127	0.001	3.96E-08
rs4746746	10	64699981	T	C	0.126	0.001	4.81E-08
rs6479846	10	64655471	C	A	0.126	0.001	4.87E-08
rs6479848	10	64659101	C	T	0.126	0.001	4.94E-08
rs6479860	10	64745865	T	C	0.127	0.001	4.58E-08
rs7100204	10	64640543	C	T	0.127	0.001	4.02E-08
rs7100320	10	64640594	C	T	0.129	0.001	2.91E-08
rs7665570	4	42152351	A	C	0.123	0.001	4.34E-08
**Vitamin/mineral supplement**							
rs10005662	4	42178259	T	C	-0.042	0.004	2.84E-06
rs10010204	4	42162182	C	G	-0.042	0.004	2.31E-06
rs10034294	4	42187640	T	C	-0.051	0.005	5.80E-07
rs10459544	14	74419191	G	C	-0.045	0.005	3.72E-06
rs11159045	14	74378984	T	C	-0.045	0.005	4.85E-06
rs11559994	12	40336494	A	G	-0.034	0.004	4.50E-06
rs12590001	14	74368920	C	T	-0.045	0.005	4.80E-06
rs1963798	14	74372862	C	T	-0.045	0.005	4.79E-06
rs2079632	14	74421212	G	C	-0.046	0.005	3.03E-06
rs28591364	4	42174012	G	T	-0.042	0.004	2.73E-06
rs28668005	4	42174395	T	C	-0.042	0.004	2.99E-06
rs28687702	4	42181516	T	G	-0.042	0.004	3.00E-06
rs28719870	4	42157823	G	A	-0.052	0.005	1.51E-07
rs34902783	4	42184017	C	A	-0.042	0.004	2.84E-06
rs35079923	4	42184622	T	A	-0.042	0.004	2.98E-06
rs4293573	2	161877629	T	G	0.032	0.003	4.62E-06
rs4299622	4	42164386	G	A	-0.042	0.004	2.55E-06
rs486112	10	43243510	G	A	-0.042	0.004	4.40E-06
rs4903163	14	74451171	C	T	-0.046	0.005	3.17E-06
rs493230	10	43247120	A	G	-0.042	0.004	4.55E-06
rs507485	10	43250110	C	A	-0.059	0.005	3.04E-07
rs538429	10	43251146	T	C	-0.042	0.004	4.86E-06
rs56087460	4	42171632	A	G	-0.042	0.004	2.74E-06
rs618687	10	43232419	G	A	-0.042	0.004	3.98E-06
rs671429	10	43248258	C	T	-0.042	0.004	4.28E-06
rs677749	10	43243001	C	A	-0.042	0.004	3.98E-06
rs6811916	4	42183856	C	T	-0.042	0.004	2.79E-06
rs6828811	4	42161066	C	T	-0.052	0.005	1.23E-07
rs6857720	4	42158529	A	T	-0.051	0.005	2.43E-07
rs7141392	14	74428286	G	A	-0.046	0.005	3.17E-06
rs71608101	4	42168329	T	C	-0.042	0.004	2.57E-06
rs71608102	4	42168399	T	G	-0.042	0.004	2.55E-06
rs71648648	1	241472176	A	G	-0.077	0.008	2.16E-06
rs7665570	4	42152351	A	C	-0.042	0.004	4.22E-06
rs7960023	12	40329432	C	A	-0.034	0.004	3.94E-06
rs8016802	14	74462213	G	A	-0.045	0.005	4.64E-06

SNP = single nucleotide polymorphism; CHR: chromosome; POS: position; SE: standard error.

Supplemental Table S2. Potential confounders of SNPs for chondroitin intake under the condition of $P < 5 \times 10^{-8}$ in the PhenoScanner

Exposure	SNP	Trait
Chondroitin	rs10246938a	Mineral and other dietary supplements: fish oil<br>Average weekly beer plus cider intake
	rs10247195a	Mineral and other dietary supplements: fish oil<br>Average weekly beer plus cider intake
	rs10268402a	Mineral and other dietary supplements: fish oil<br>Average weekly beer plus cider intake
	rs10282264a	Mineral and other dietary supplements: fish oil<br>Average weekly beer plus cider intake
	rs11240229b	Qualifications: college or university degree<br>Years of educational attainment<br>Mineral and other dietary supplements: fish oil<br>Years of educational attainment in females
	rs11584700b	College completion<br>College completion females<br>Educational attainment<br>Educational attainment females<br>Educational attainment<br>Mineral and other dietary supplements: fish oil<br>Qualifications: college or university degree<br>College completion<br>Years of educational attainment in females<br>Years of educational attainment
	rs11588857b	College completion<br>Cognitive ability multi trait analysis<br>Educational attainment years of education<br>Intelligence multi trait analysis<br>Mineral and other dietary supplements: fish oil<br>Qualifications: college or university degree<br>College completion<br>Years of educational attainment in females<br>Years of educational attainment
	rs12040459b	Mineral and other dietary supplements: fish oil<br>Qualifications: college or university degree<br>Years of educational attainment in females<br>Years of educational attainment
	rs12040520b	Mineral and other dietary supplements: fish oil<br>Qualifications: college or university degree<br>Years of educational attainment in females<br>Years of educational attainment
	rs12044599b	Mineral and other dietary supplements: fish oil<br>Qualifications: college or university degree<br>Years of educational attainment in females<br>Years of educational attainment
	rs12046747b	College completion<br>Mineral and other dietary supplements: fish oil<br>Qualifications: college or university degree<br>College completion<br>Years of educational attainment in females

		Years of educational attainment
	rs16853666	Mineral and other dietary supplements: fish oil
	rs16854023b	College completion<br>Mineral and other dietary supplements: fish oil<br>Qualifications: college or university degree<br>College completion<br>Years of educational attainment in females<br>Years of educational attainment
	rs2098112a	Mineral and other dietary supplements: fish oil<br>Average weekly beer plus cider intake<br>Crohns disease
	rs2533123a	Mineral and other dietary supplements: fish oil<br>Average weekly beer plus cider intake
	rs2533125a	Mineral and other dietary supplements: fish oil<br>Average weekly beer plus cider intake
	rs2533126a	Mineral and other dietary supplements: fish oil<br>Average weekly beer plus cider intake
	rs2533128a	Mineral and other dietary supplements: fish oil<br>Average weekly beer plus cider intake
	rs2533145a	Mineral and other dietary supplements: fish oil<br>Average weekly beer plus cider intake
	rs2533148a	Mineral and other dietary supplements: fish oil<br>Average weekly beer plus cider intake<br>Crohns disease
	rs2533273a	Mineral and other dietary supplements: fish oil<br>Average weekly beer plus cider intake
	rs2622166a	Mineral and other dietary supplements: fish oil<br>Average weekly beer plus cider intake
	rs2622170a	Mineral and other dietary supplements: fish oil<br>Average weekly beer plus cider intake
	rs2622171a	Mineral and other dietary supplements: fish oil<br>Average weekly beer plus cider intake
	rs2622217a	Mineral and other dietary supplements: fish oil<br>Average weekly beer plus cider intake
	rs2622225a	Mineral and other dietary supplements: fish oil<br>Average weekly beer plus cider intake
	rs2622226a	Mineral and other dietary supplements: fish oil<br>Average weekly beer plus cider intake
	rs2907674a	Mineral and other dietary supplements: fish oil<br>Average weekly beer plus cider intake
	rs3747630b	College completion<br>Mineral and other dietary supplements: fish oil<br>Qualifications: college or university degree<br>College completion<br>Years of educational attainment
	rs3789044b	Mineral and other dietary supplements: fish oil<br>Qualifications: college or university degree<br>Years of educational attainment in females<br>Years of educational attainment
	rs3789044b	College completion<br>Educational attainment<br>Mineral and other dietary supplements: fish oil<br>Qualifications: college or university degree<br>College completion

		Years of educational attainment in females<br>Years of educational attainment
	rs55678522b	Mineral and other dietary supplements: fish oil<br>Qualifications: college or university degree<br>Years of educational attainment in females<br>Years of educational attainment
	rs55979051b	Mineral and other dietary supplements: fish oil<br>Qualifications: college or university degree<br>Years of educational attainment in females<br>Years of educational attainment
	rs61817482b	Mineral and other dietary supplements: fish oil<br>Qualifications: college or university degree<br>Years of educational attainment in females<br>Years of educational attainment
	rs6958769a	Mineral and other dietary supplements: fish oil<br>Average weekly beer plus cider intake
	rs73488191a	Mineral and other dietary supplements: fish oil<br>Average weekly beer plus cider intake

[a] SNPs are associated with potential confounders "beer plus cider intake", while [b] SNPs are associated with potential confounders "high educational status"

Supplemental Table S3. Pleiotropy-robust MR results for dietary supplement intake and kidney function

Exposure	Analysis	[a]Outcome	MR-Egger intercept P	MR methods	eGFR change beta (%)	Standard error (%)	P value
Chondroitin	Main	Creatinine-based log-eGFR (CKDGen)	0.013	MR-Egger	-0.012	0.004	0.007
				Weighed median	-0.001	0.001	0.255
	Validation	Creatinine-based log-eGFR (CKDGen+UKB)	0.2	MR-Egger	-0.572	0.297	0.026
				Weighed median	-0.159	0.044	3E-04
Glucosamine	Main	Creatinine-based log-eGFR (CKDGen)	0.419	MR-Egger	-0.277	0.741	0.357
				Weighed median	-0.182	0.121	0.132
	Validation	Creatinine-based log-eGFR (CKDGen+UKB)	0.02	MR-Egger	1.682	0.434	0
				Weighed median	0.551	0.098	1E-08
Vitamin/ mineral supplement intake	Main	Creatinine-based log-eGFR (CKDGen)	0.077	MR-Egger	2.129	1.256	0.036
				Weighed median	1.239	0.262	2E-06
	Validation	Creatinine-based log-eGFR (CKDGen+UKB)	0.612	MR-Egger	0.201	1.001	0.416
				Weighed median	1.429	0.217	4E-11

MR = Mendelian randomization; eGFR = estimated glomerular filtration rate
[a] For main and validation datasets, meta-analysis of creatinine-based log-eGFR from CKDGen and meta-analysis of creatinine-based log-eGFR from the CKDGen and UKB were used as outcome summary statistics, respectively.[1, 2]
The MR estimates were converted into the degree of change (percentage [standard error]) of log-transformed eGFR to facilitate interpretation.

Supplemental Table S4. Sensitivity analyses of chondroitin intake and kidney function after exclusion of SNPs associated with potential confounders identified in PhenoScanner

[a]Outcome	MR methods	eGFR change beta (%)	Standard error (%)	P value
Exclusion of SNPS associated with beer plus cider intake ([a] SNPs)				
Creatinine-based log-eGFR (CKDGen)	MR-IVW	-0.247	0.040	7E-10
Creatinine-based log-eGFR (CKDGen+UKB)	MR-IVW	-0.217	0.026	3E-17
Exclusion of SNPS associated with high educational status ([b] SNPs)				
Creatinine-based log-eGFR (CKDGen)	MR-IVW	1E-05	0.026	0.976
Creatinine-based log-eGFR (CKDGen+UKB)	MR-IVW	-0.135	0.011	9E-36

SNP = single nucleotide polymorphism; MR = Mendelian randomization; eGFR = estimated glomerular filtration rate; MR-IVW = multiplicative random-effects inverse variance-weighted

[a] SNPs are associated with potential confounders “beer plus cider intake”, while [b] SNPs are associated with potential confounders “high educational status”

[a] Sensitivity analyses were performed with both main and validation datasets, which include meta-analysis of creatinine-based log-eGFR from CKDGen and meta-analysis of creatinine-based log-eGFR from the CKDGen and UKB.[1, 2]

Supplemental References

1. Stanzick KJ, Li Y, Schlosser P, *et al.* Discovery and prioritization of variants and genes for kidney function in >1.2 million individuals. *Nat Commun* 2021; **12:** 4350.

2. Wuttke M, Li Y, Li M, *et al.* A catalog of genetic loci associated with kidney function from analyses of a million individuals. *Nat Genet* 2019; **51:** 957-972.
